# National-Level Schoolwork Pressure, Family Structure, Internet Use, and Obesity as Drivers of Time Trends in Adolescent Psychological Complaints Between 2002 and 2018

**DOI:** 10.1007/s10964-023-01800-y

**Published:** 2023-06-22

**Authors:** M. Boer, A. Cosma, J. M. Twenge, J. Inchley, H. Jeriček Klanšček, G. W. J. M. Stevens

**Affiliations:** 1 Department of Interdisciplinary Social Science, Utrecht University, Utrecht, the Netherlands; 2 Department of Sociology, Trinity College Dublin, Dublin, Ireland; 3 Department of Psychology, San Diego State University, San Diego, CA USA; 4 MRC/CSO Social and Public Health Sciences Unit, University of Glasgow, Glasgow, UK; 5 National Institute of Public Health, Ljubljana, Slovenia

**Keywords:** Mental health problems, Trends, Mid-adolescence, Gender, Health Behavior in School-aged Children (HBSC)

## Abstract

Little is known about societal processes that contribute to changes in adolescent mental health problems. This study aims to fill this gap using data from the Health Behavior in School-aged Children study between 2002 and 2018 (*n*_countries_ = 43, *n*_individuals_ = 680,269, *M*_age_ = 14.52 (*SD* = 1.06), 51.04% female), supplemented with other international data. National-level psychological complaints increased more strongly among girls than boys. National-level schoolwork pressure, single-parent households, time spent on internet, and obesity were generally rising. In both boys’ and girls’ samples, increases in national-level schoolwork pressure, obesity, and time spent on internet use were independently associated with increases national-level psychological complaints. However, national-level obesity and psychological complaints were more strongly related among girls than boys. Results highlight the potential impact of societal-level processes on adolescent mental health problems.

## Introduction

In recent years, there has been widespread societal concern about rises in mental health problems among early-mid adolescents, especially internalizing problems (e.g., anxiety, depression) (Bor et al., 2014). Indeed, multiple studies found an increase in such mental health problems since the beginning of the 21^st^ century in the United States and several European countries (Collishaw, 2015; Fink et al., 2015; Högberg et al., 2020; Kim & Hagquist, 2018; Potrebny et al., 2019; Twenge et al., 2018). Yet, some internationally comparative studies indicated that time trends in adolescent mental health vary considerably across countries (Cosma et al., 2020; Whitehead et al., 2017). Currently, little is known about the national-level processes associated with these diverse time trends in adolescent mental health. Identifying such trends not only adds to the scientific research on the development of adolescent mental health problems, but can also be crucial to the development of national-level policies. Therefore, using data from the Health Behavior in School-aged Children (HBSC) study from 2002, 2006, 2010, 2014, and 2018 across 43 countries, the current study investigates whether and to what extent several societal changes at the national level are associated with national-level changes in mental health problems among adolescents. Although adolescence covers the age span 10 to 24, this study focuses on the mid-adolescence years, when mental health problems seem to reach a peak (Inchley et al. 2020; Solmi et al. 2022).

### Societal Changes Associated With National-Level Changes in Mental Health Problems

Internationally comparative research on time trends in mental health problems is scarce, even more so when it accounts to processes associated with these trends. Previous research either used theoretical reasoning to explain different trends across countries (e.g., by using Lazarus and Folkman’s stress model and focusing on increases in unemployment and decreases in school achievement) (Bremberg, 2015; Ottová-Jordan et al., 2015), or focused on factors at the individual level to explain time trends in mental health problems (e.g., individual schoolwork pressure and school wellbeing) (Cosma et al., 2020; Högberg, 2021; Marquez et al., 2022). Notwithstanding the relevance of previous approaches, these are less able to assess broad societal trends that may affect the mental health of whole cohorts of adolescents in particular national contexts (Högberg, 2021), which is the focus of this study. After reviewing the existing literature, four factors were identified that have been studied extensively in relation to adolescent mental health at the individual level, but could also emerge as societal drivers of time trends in adolescent mental health problems.

First, research using HBSC-data indicates that adolescents’ perceived *schoolwork pressure* increased in 21 countries between 2014 and 2018, while it decreased in only three countries and remained the same in 17 countries (Inchley et al., 2020). This phenomenon may be due to the importance placed on the attainment of a high education level, which has become increasingly salient in some societies (Curran & Hill, 2019), but also due to an increase in parental demands and criticism (Curran & Hill, 2022). Second, *family structures* have changed over recent decades, with many Western countries showing an increase in households headed by single mothers (Kollmeyer, 2013). Correspondingly, in 26 countries participating in the HBSC study, the proportion of adolescents living with both parents in one house decreased between 1994 and 2018, with decreases ranging from 3.2 to 17.1 percentage points across countries (Zaborskis et al., 2022). This societal change is more apparent in some than in other countries and may be related to cross-national variation in increasing divorce rates related to different developments, such as women’s labor force participation and norms regarding gender equality across countries (C. T. L. Wang & Schofer, 2018). Third, globally, *internet use* has increased considerably in the past decades, although this increase also varied substantially across countries (World Bank, 2022). The improved accessibility of the internet (OECD, 2019a), together with the increasing popularity of social media among adolescents (Anderson & Smith, 2021), have probably contributed to this development. Fourth, research on childhood and adolescent *obesity* in 1980–2010 shows that while in some parts of Europe the prevalence of obesity has been stable since 2000, other parts of Europe have shown increases in prevalence (Bentham et al., 2017). Also, a recent report indicates that in a significant number of countries participating in the HBSC study there was an increase in adolescent obesity rates (Inchley et al., 2017). Changes in adolescents’ energy intake, physical activity and having parents who are overweight may have contributed to these trends in obesity (Livingstone, 2001).

There is limited research connecting these societal developments with trends in mental health problems across countries. Nevertheless, it is conceivable that societal processes related to changes in schoolwork pressure, family structures, internet use, and obesity, have put pressure on adolescents in various manners: a pressure to succeed academically, romantically, socially, and physically. More specifically, societies where schoolwork pressure is normative may create environments with a strong emphasis on academic achievements (Högberg, 2021). Also, growing up in societies with higher levels of family breakdown could make adolescents more aware of the vulnerability of romantic relationships and may weaken important social support systems. Further, increasing levels of internet use imply increasing levels of social media use, especially since the introduction of the smartphone (Twenge et al., 2018). The increasing popularity of social media may shape cultures where monitoring peers online becomes an integral part of social relationships, which could increase adolescents’ sensitivity to engaging in social comparisons, such as perceiving the lives of peers as superior to their own (Verduyn et al., 2017). Finally, recent increases in obesity rates have been found to co-occur with increases in weight stigma prevalence, that is, negative attitudes and beliefs about people with higher weight (Papadopoulos & Brennan, 2015). In societies where obesity is salient and where there is weight-based stigmatization, adolescents may be more aware of physical appearance and differences in this regard, affecting their own body image (Dzielska et al., 2020). These different societal phenomena may pressure adolescents to succeed in several important life domains, which could induce inner conflicts between their actual and aspired self, ultimately leading to mental health problems (Curran & Hill, 2022).

### Gender Differences in the National-Level Drivers of Time Trends in Adolescent Mental Health Problems

International research suggests that increases in mental health problems, especially emotional problems, in the last decades were stronger among girls than boys (Bor et al., 2014), and that these differences peak in adolescence (Salk et al., 2017). One of the explanations for this phenomenon may be that girls’ biological, affective, and cognitive characteristics make them more likely to experience stress as a response to the issues they encounter in their lives and to exhibit greater levels of mental health problems than boys in response to these stressors (Hankin et al., 2007). Correspondingly, research shows that, at the individual level, girls for instance more often perceive schoolwork pressure than boys (Cosma et al., 2020) and have a more negative image of their own bodies (Whitehead et al., 2017). Also, studies show that individual-level schoolwork pressure, family structure, internet use, and obesity were more strongly linked to mental health problems of girls than of boys (De Looze et al., 2020; Marmorstein et al., 2014; Tullius et al., 2022; Twenge & Farley, 2021). Therefore, this study also investigates gender differences in the aforementioned societal stressors and in their associations with mental health problems in adolescents.

## Current Study

The potential impact of national-level processes on trends in adolescent mental health problems have been understudied. Using data from 43 countries that participated in the HBSC study, the objective of the study was to fill this gap by investigating whether changes in country-level (a) schoolwork pressure, (b) family structure, (c) internet use, and (d) adolescent obesity across 2002–2018 were associated with changes in country-level psychological complaints among adolescents in the same time period. This study focuses on 13- and 15-year-old adolescents, because mental health problems, high schoolwork pressure, and intensive internet use have been found to be more prevalent among somewhat older adolescents. Furthermore, differences in these associations between boys and girls are investigated. It was hypothesized that rises in schoolwork pressure, internet use, and adolescent obesity, and declines in the two-parent family structure at the national level, were associated with national-level increases in psychological complaints in adolescents. These associations were expected to be stronger in girls than in boys.

## Methods

### Data

The main data source for the present study is the HBSC study. The study includes nationally representative repeated individual-level cross-sectional data from adolescents from the European region and North America collected every four years since 1982 in collaboration with the World Health Organization (WHO) Regional Office for Europe. The aim of the HBSC study is to monitor and understand the health (behaviors) and wellbeing of adolescents aged 11, 13, and 15 in their social context. The survey questions were subjected to a translation and back-translation procedure following the international HBSC research protocol (Inchley et al., 2018). In each survey cycle, countries strictly followed the sampling method and procedures according to the protocol, which prescribed sampling of adolescents through randomly selected schools and/or classes (Inchley et al., 2018). As such, the data had a hierarchical structure with four levels, with *adolescents* on the first level, *school (classes)* on the second level, *country-years* (i.e., years within countries) on the third level, and *countries* on the fourth level. Participation was voluntary and anonymous. Parents of respondents provided active or passive consent, depending on the country. Surveys were completed using digital or paper-and-pencil self-completion in classrooms during school hours. Ethical approval of the study procedures was obtained from the institutional ethic committees from each participating country (Inchley et al., 2018). The HBSC data were supplemented with data from the Programme for International Student Assessment (PISA), WHO’s Global Health Observatory, and World Bank, based on country, year and gender. For England, Scotland, and Wales, United Kingdom-level data were used where information was not available at the national level. Similarly, Belgian data were used for both Flanders and Wallonia where regional level data were not available.

The original international HBSC datafile counted 1,356,191 participants from 47 countries and two Belgium regions (French and Flemish) (HBSC, 2021). For consistency, these two regions are referred to as countries. Data from survey cycles 2002, 2006, 2010, 2014, and 2018 were selected (*n*_individuals_ = 1,045,745, *n*_countries_ = 48). Next, data from countries that participated in at least two survey cycles were selected (*n*_individuals_ = 1,028,116, *n*_countries_ = 44), which was required in order to study change over time. Subsequently, 13- and 15-year-olds were selected (*n* = 684,024, *n*_countries_ = 44). Finally, data from one country were removed (Greenland), because neither of the external data sources included data for this country, resulting in a sample of 680,269 adolescents (*n*_countries_ = 43, 51.04% girl, 48.72% 15-year-olds).

### Measures

#### Psychological complaints (HBSC)

The outcome measure was assessed using a four-item subscale from the HBSC Symptoms Checklist (Heinz et al., 2022). Respondents were asked “In the last 6 months, how often have you had the following…”, followed by “feeling low”, “irritability or bad temper”, “feeling nervous”, and “difficulties getting to sleep”. Responses ranged from 1 = *about every day* to 5 = *rarely or never*. The subscale has shown good internal reliability and appropriate construct validity with other mental health indicators among adolescents (Gariepy et al., 2016; Heinz et al., 2022). In the present sample, Cronbach’s alpha for the four items was 0.753. Scores on the four items were rescaled and summed, such that higher values denoted higher levels of psychological complaints. To indicate adolescents with high levels of psychological complaints, dummy variable was created based on the 80^th^ percentile of the sum-score among 13- and 15-year-olds between 2002 and 2018, which referred to a sum-score of at least 13 (1 = *psychological complaints > 80*^*th*^ *centile* and 0 = *psychological complaints < 80*^*th*^ *centile*). This cut-off is in line with cut-offs of other measures of mental health problems (Dierker et al., 2001; Goodman et al., 1998).

### Explanatory variables

#### Proportion with high schoolwork pressure (HBSC)

Respondents were asked “How pressured do you feel by the schoolwork you have to do?”, with responses 1 = *not at all*, 2 = *a little*, 3 = *some*, and 4 = *a lot*. This measure has been used extensively to study cross-national trends in adolescent schoolwork pressure (Cosma et al., 2020; Klinger et al., 2015; Löfstedt et al., 2020). To indicate adolescents experiencing high schoolwork pressure, a dummy variable was created (1 = *some / a lot schoolwork pressure* and 0 = *not at all / a little schoolwork pressure*), which corresponds to the operationalization of schoolwork pressure in the international HBSC reports (Inchley et al., 2016, 2020). Subsequently, the proportion of boys and girls reporting high schoolwork pressure for each year within each country was computed.

#### Proportion living with both parents (HBSC)

Respondents were asked to “Please answer this question for the home where you live all or most of the time and tick the people who live there”, whereby respondents could select *mother*, *father*, *stepmother (or father’s girlfriend/partner)*, *stepfather (or mother’s boyfriend/partner)*, *I live in a foster home or children’s home*, and an open answer category *someone or somewhere else (e.g., siblings, grandparents)*. A dummy variable was created indicating whether respondents lived with both parents in their main home (1 = *mother and father in main home* and 0 = *other*). Subsequently, the proportion of boys and girls living with both parents for each year within each country was computed.

#### Average time spent on internet (PISA)

Data on adolescents’ average time spent on internet were derived from individual-level cross-sectional data from the PISA study conducted in 2012, 2015 and 2018 (OECD, 2019b)^1^. Respondents were asked: “On a typical weekday, for how long do you use the Internet outside of school?” with responses recoded to reflect hours per day: 0 = *no time*, 0.25 = *1*–*30* *min per day*, 0.75 = *31*–*60* *min per day*, 1.5 = *between 1 and 2* *h per day*, 3 = *between 2 and 4* *h per day*, 5 = *between 4 and 6* *h per day*, and 7 = *more than 6* *h per day*. Subsequently, the average number of hours boys and girls spent on internet use were computed by year and country. These data were linked to the HBSC data based on country, year of participation, and gender. Because the HBSC and PISA survey cycles did not run entirely parallel, the HBSC 2010 data were linked with 2012 PISA data, the HBSC 2014 data with 2015 PISA data, and the HBSC 2018 with 2018 PISA data.

#### Proportion with obesity (WHO)

Data on obesity were taken from the WHO’s Global Health Observatory (WHO, 2021)^2^. The indicator represents the proportion of adolescents aged 10 to 19 with a Body Mass Index (BMI) of two standard deviations or more above the median of the WHO gender- and age-specific growth references for children and adolescents (Abarca-Gómez et al., 2017). The dataset was compiled using data sources on weight and height excluding sources based on solely self-reported weight and height, that are typically subject to bias (Abarca-Gómez et al., 2017). These data were linked to the HBSC data based on country, year, and gender. The HBSC 2002, 2006, 2010, and 2014 data were linked with WHO obesity data from the corresponding years, whereas HBSC 2018 data were linked with 2016 WHO obesity data, as more recent measures of obesity were not available.

### Control variables

#### Demographic characteristics (HBSC)

*Gender* was assessed by asking respondents whether they are boy or girl (1 = *girl*, 0 = *boy*). Respondents’ *age* was calculated based on their birth date and the day of survey completion. A dummy was created to indicate age (1 = *15-year-old* and 0 = *13-year-old*). *Family affluence* was measured using the 4-item Family Affluence Scale (Currie et al., 2008), which is a measure of socioeconomic status. It examined adolescents’ material assets within the household, such as the number of family holidays spent abroad in past year (0 = *not at all*, 1 = *once*, 2 = *twice*, and 3 = *more than twice*). A sum-score was computed such that higher values indicated higher family affluence.

#### Income inequality (World Bank)

*Income inequality* was indicated by the Gini index obtained from household survey data accessed through World Bank Open Data (Worldbank, 2021b). It ranges from 0 to 100, whereby a value of 0 corresponded to maximum income equality and a value of 100 to maximal income inequality. These data were linked to the HBSC data based on country and year. If data on income inequality were unavailable for a specific HBSC survey cycle within a particular country (15%), the available data of the year and country closest to the missing observation were used.

#### Economic performance (World Bank)

*Economic performance* was indicated by the Gross Domestic Product (GDP) obtained from national accounts data accessed through World Bank Open Data (Worldbank, 2021a). GDP referred to the GDP divided by the midyear population in constant 2010 U.S. dollars. The GDP values were divided by 10.000 to decrease variance, which was necessary to overcome estimation problems in the analyses. These data were linked to the HBSC data based on country and year. In all countries, World Bank data on economic growth were available for all five HBSC survey cycles.

### Analyses

#### Preliminary analysis

In the first step, the country means and proportions of the study variables were computed by gender and survey year. Gender differences were examined using *t*-tests. Cohen’s *D* was used to calculate effect sizes of the gender differences (0.2 = small, 0.5 = moderate, 0.8 = large (Cohen, 1988)). In addition, bivariate correlations were calculated at the country-year level between the *cluster mean centered* study variables by gender (0.1 = small, 0.3 = moderate, 0.5 = large (Cohen, 1988)). Cluster mean centering means that the country-year-level data was centered by their respective country-level mean (Fairbrother, 2014). Correlations between the cluster mean centered study variables and *time* were computed, whereby time was operationalized as a continuous variable ranging from 0 to 4 (i.e., from 2002 to 2018, respectively).

### Main analysis

#### Analytical model

Given that the outcome was dichotomous, this study used three-level logistic regression modeling with Maximum Likelihood estimation to study the research questions using the *glmer-*function from the *lme4-*package in R version 4.2.2 (Bates et al., 2015; R Core Team, 2022). Individuals *i* were nested within country-years *t*, which were nested within countries *c* (Schmidt-Catran & Fairbrother, 2016). The school level was not considered because this yielded convergence problems in the analyses. Associations at the country-year level denote the associations between different trends, which aim to answer the research questions. The predictors at this level were the countries’ cluster-mean centered yearly (1) proportion of adolescents reporting high schoolwork pressure, (2) proportion of adolescents living with both parents, (3) average hours of internet use, and (4) proportion of adolescents with obesity, controlling for (5) income inequality, and (6) economic performance. The analyses controlled for age and family affluence at the individual level, as they can explain variance at higher levels (Hox, 2010b). Also, the analyses controlled for the country means across years in high schoolwork pressure, family structure, internet use, obesity, income inequality and economic performance at the country level to disentangle within- and between-country effects (Fairbrother, 2014). Income inequality and economic performance were added as control variables, as previous research indicated that higher per person income and income inequality at the national level were associated with lower psychological symptoms (Elgar et al., 2015).

#### Modelling steps

In the first step, the intra class correlations (ICCs) of psychological complaints at the country-year level were computed. The ICC denotes the extent to which psychological complaints varied over time within a country, relative to the total variance in psychological complaints (i.e., at the individual, country-year, and country level). Next, a model with the control variables (Model A) was fitted. Subsequently, this model was extended with one of the main predictors, that is, schoolwork pressure, family structure, internet hours, or obesity (Model B). To examine whether trends in schoolwork pressure, family structure, internet use, and obesity potentially confound each other in their effects on trends in psychological complaints, a final model was tested that included all four predictors simultaneously. All models were conducted for boys and girls separately^3^. Details regarding the model specification can be found in the Supplementary (Fig. S1).

The significance of the tested associations was based on *p*-values estimates (< 0.05), as well as the change in model fit of Model B relative to Model A. Model fit was evaluated based on the Akaike Information Criterion (AIC), Bayesian Information Criterion (BIC), and the deviance, whereby lower values in these indices indicated better model fit (Hox, 2010a). The change in the deviance was tested using a chi-square test (*p* < 0.05). To investigate whether the associations between the main predictors and psychological complaints differed by gender, the *z*-scores of the estimate differences between boys and girls were used (Paternoster et al., 1998). Scripts of the data handling and analyses are publicly available via https://osf.io/5akh8/.

The sample sizes of the analysis samples differed across the models, depending on availability of data at the country-year level. Details regarding these sample sizes can be found in Table 1. In addition, countries varied in the (amount of) years they participated in the survey and therefore differed in data availability. However, the analytical approach does not require an equal number of observations at the country-year level. Across the analysis samples, missing data percentages on family affluence and psychological complaints were low (between 4.86 and 7.76%). Adolescents with missing data were excluded. In general, across all analysis samples (Table 1), there were very small differences in psychological complaints, age, and family affluence between included and excluded adolescents (max. Cramer’s *V* = 0.04).

**Table 1:** Data selection

Analysis sample	Years	Sex	*n* complete country-year-level data^a^			*n* complete country-year-level data + individual variables^b^			% missing
			L1	L2	L3	L1	L2	L3	
Model 1 (schoolwork pressure)	2002–2018	Boys	318,494	188	43	294,426	188	43	7.56
		Girls	332,010	188	43	315,881	188	43	4.86
Model 2 (family structure)	2002–2018	Boys	315,212	186	43	291,338	186	43	7.57
		Girls	328,664	186	43	312,667	186	43	4.87
Model 3 (time spent on internet)	2010–2018	Boys	142,232	81	31	131,287	81	31	7.70
		Girls	146,784	81	31	139,081	81	31	5.25
Model 4 (obesity)	2002–2018	Boys	318,494	188	43	294,426	188	43	7.56
		Girls	332,010	188	43	315,881	188	43	4.86
Model 5 (final)	2010–2018	Boys	137,178	78	30	126,538	78	30	7.76
		Girls	141,538	78	30	134,027	78	30	5.31

L1 = level 1, i.e., individuals; L2 = level 2, i.e., country-years or years within countries; L3 = level 3, i.e., countries

^a^cases from countries where data on the proportion of adolescents with high psychological complaints, the respective predictor(s), and the control variables income inequality and economic performance at the country-year level were all available within the same year for at least two years

^b^cases from countries where data on the proportion of adolescents with high psychological complaints, the respective predictor(s), and the control variables income inequality and economic performance at the country-year level were all available within the same year for at least two years AND where data on the individual level variables on high psychological complaints, family affluence, and age were complete

#### Interpretation of effects

Coefficients from (multilevel) logistic regression cannot be transformed into standardized effects. To facilitate comparison of the effects of the four predictors, the proportions of adolescents with high psychological complaints were estimated by standardized scores of the predictors using the *ggeffects*-package for Rstudio, based on the their logit estimates from the models (Lüdecke, 2018). Also, the models used the cluster mean centered values in the analyses, as recommended for studying relations between trends (Fairbrother, 2014). Given these model specifications, associations denote, for example, the extent to which yearly increases in the average time spent on internet use relative to the countries’ average level of internet use co-occurred with yearly increases in the percentage of adolescents with high psychological complaints relative to the countries’ average percentage with psychological complaints.

### Sensitivity analyses

To capture country-level effects that go *beyond* effects at the individual level, that is, that reflect societal level effects rather than an aggregation of effects operating at the individual level, adding individual-level covariates is important (Brincks et al., 2017). Therefore, it was tested whether controlling for schoolwork pressure and living with both parents at the individual level impacted the effects of the proportion of schoolwork pressure and living with both parents at the country-year level. It was also tested whether controlling for self-report obesity as assessed in the HBSC study impacted the effect of the proportion with obesity at the country-year level. Self-reported obesity was identified based on adolescents’ reported weight and height, and gender- and age-specific thresholds for obesity (WHO, 2006). Although self-report weight and height by adolescents are less valid than direct assessments of an adolescent’s weight and height (Abarca-Gómez et al., 2017), country-year-level aggregated self-report obesity and WHO’s measure of obesity based on direct assessments correlated strongly (girls *r* = 0.697, boys *r* = 0.741). Controlling for internet use at the individual level was not possible, because the HBSC study does not include an internet use related measure that has been repeatedly assessed within the different fieldwork periods. In addition, sensitivity to the selected cut-off value for psychological complaints was examined, by repeating the analyses using cut-off values based on the 70^th^ and 90^th^ centile next to the 80^th^ centile.

## Results

### Preliminary Analyses

#### Descriptive statistics

Across countries, the proportion of girls with high psychological complaints was much higher than that of boys (Table 2; Cohen’s *D* = 1.70). Also, the proportion of adolescents reporting high schoolwork pressure was higher among girls than among boys (Cohen’s *D* = 0.57). The proportion of girls with obesity was substantially lower than that of boys (Cohen’s *D* = 0.99). The average level of time spent on internet and proportion living with both parents did not vary by gender. The proportions of boys and girls with high psychological complaints, high schoolwork pressure, obesity, and the average time spent on internet, fluctuated over time, but were generally on the rise, while the proportions of adolescents living with both parents were generally declining. However, trends differed by country (Supplementary Figs. S2-S8).

**Table 2:** Descriptive statistics uncentered country-year-level variables

	Total					2002		2006		2010		2014		2018	
	*n*	Mean	*SD*	Min.	Max.	*n*	Mean	*n*	Mean	*n*	Mean	*n*	Mean	*n*	Mean
**Boys**															
Proportion with high psychological complaints	188	0.154	0.062	0.05	0.44	30	0.140	39	0.152	38	0.145	40	0.147	41	0.181
Proportion with schoolwork pressure	188	0.365	0.116	0.14	0.68	30	0.372	39	0.382	38	0.349	40	0.345	41	0.380
Proportion living with both parents	186	0.744	0.082	0.41	0.94	30	0.777	39	0.731	37	0.742	40	0.751	40	0.727
Time spent in internet (hours)	81	3.267	0.609	1.48	4.44					23	2.600	29	3.318	29	3.744
Proportion with obesity	188	0.081	0.029	0.03	0.21	30	0.069	39	0.073	38	0.079	40	0.086	41	0.092
**Girls**															
Proportion with high psychological complaints	188	0.282	0.087	0.10	0.62	30	0.241	39	0.265	38	0.253	40	0.297	41	0.342
Proportion with schoolwork pressure	188	0.439	0.138	0.17	0.79	30	0.427	39	0.437	38	0.404	40	0.435	41	0.485
Proportion living with both parents	186	0.732	0.089	0.46	0.93	30	0.761	39	0.722	37	0.732	40	0.737	40	0.715
Time spent in internet (hours)	81	3.147	0.687	1.01	4.33					23	2.283	29	3.291	29	3.687
Proportion with obesity	188	0.053	0.026	0.02	0.18	30	0.048	39	0.050	38	0.054	40	0.055	41	0.059
**Not gender specific**															
Income inequality	188	32.011	4.753	22.60	42.80	30	32.527	39	32.308	38	31.750	40	31.910	41	31.690
Economic performance	188	3.333	2.281	0.21	11.07	30	2.951	39	3.388	38	3.404	40	3.239	41	3.586

*n* sample size, *SD* standard deviation, *Min.* minimum, *Max.* maximum

The vast majority of countries observed increases between 2002 and 2018 in psychological problems in both boys and girls, but the strength of this increase varied substantially across countries (e.g., from 0.239 to 0.299 among Croatian girls, compared to from 0.217 to 0.376 among Scottish girls; Supplementary Fig. S2). Also in most countries the proportion of girls with high schoolwork pressure increased between 2002 and 2018, although again the magnitude of this increase differed across countries (e.g., from 0.257 to 0.292 in Austria, compared to from 0.204 to 0.453 in the Netherlands; Supplementary Fig. S3). Patterns regarding time trends in boys’ schoolwork pressure were somewhat more mixed: although in the majority of countries the proportion of boys with schoolwork pressure increased, in over a third of the countries a decrease in this proportion was found (Supplementary Fig. S3). Also decreases in proportions of boys and girls living with both parents in one house were found in many countries, with again sizable differences across countries (e.g., from 0.770 to 0.756 among German boys compared to from 0.768 to 0.665 among French boys between 2002 and 2018; Supplementary Fig. S4). Average time spent on internet and obesity rates increased in all countries (except for obesity among girls in Denmark), but also here, there was variation in the strength of increases across countries (Supplementary Figs. S5-S6).

#### Bivariate correlations

Table 3 shows that across countries, the proportion of girls with high psychological complaints increased strongly over time, whereas for boys this proportion increased moderately. The proportion of girls with high schoolwork pressure increased over time with small to moderate effect size, whereas for boys there was no linear trend in schoolwork pressure. Countries showed, for both boys and girls, a strong increase in the average time spent on internet and the proportion with obesity, as well as a moderate decrease in the proportion living with both parents. There were small decreases in income inequality and large increases in economic performance over time. Due to the multicollinearity between time and some of the predictors (i.e., internet hours and obesity: *r* > 0.80), the main analysis did not include time as an additional control variable.

**Table 3:** Pairwise correlations cluster mean centered country-year-level variables

	Boys	1	2	3	4	5	6	7	8
1	Time	1.000							
2	High psychological complaints	0.429***	1.000						
3	Schoolwork pressure	−0.039	0.275***	1.000					
4	Living with both parents	−0.337***	−0.303***	0.014	1.000				
5	Internet hours	0.863***	0.523***	0.180	−0.147	1.000			
6	Obesity	0.835***	0.294***	−0.132	−0.286***	0.650***	1.000		
7	Income inequality	−0.187*	−0.122	0.002	0.173*	−0.185	−0.094	1.000	
8	Economic performance	0.680***	0.378***	0.094	−0.233**	0.608***	0.576***	−0.130	1.000

	Girls	1	2	3	4	5	6	7	8
1	Time	1.000							
2	High psychological complaints	0.668***	1.000						
3	Schoolwork pressure	0.287***	0.524***	1.000					
4	Living with both parents	−0.391***	−0.325***	−0.045	1.000				
5	Internet hours	0.866***	0.725***	0.400***	−0.209	1.000			
6	Obesity	0.801***	0.505***	0.149*	−0.392***	0.644***	1.000		
7	Income inequality	−0.187*	−0.233**	−0.100	0.209**	−0.166	−0.076	1.000	
8	Economic performance	0.680***	0.551***	0.285***	−0.277***	0.540***	0.519***	−0.130	1.000

*p < 0.05; **p < 0.01; ***p < 0.001

Within countries, the higher the proportion with high schoolwork pressure, proportion with obesity, and average levels of internet use, and the lower the proportion living with both parents, the higher the proportion with high psychological complaints (Table 3). This was observed for both boys and girls. Associations varied from moderate to strong, but stronger associations were generally found for girls than for boys.

### Main Analyses

Table 4 summarizes the results from the three-level models estimating high psychological complaints. All models and their estimates can be found in the Supplementary (Tables S1-S5). For girls, the ICC at the country-year level varied between 0.022 and 0.026 across all models, which means that between 2.2 and 2.6% of all variance in high psychological complaints was explained by factors at the country-year level. For boys, this varied between 1.2 and 1.3%.

**Table 4:** Summary three-level logistic regression models, high psychological complaints

	Boys														
	Sample size			Model B country-year-level estimates			Model fit change relative to Model A				Predicted country proportions of high psychological complaints by values of predictors				
	*i*	*t*	*c*	*B*	*SE*	*p*	∆AIC	∆BIC	∆deviance	*p*	*−2 SD*^a^	*−1 SD*	0	*+ 1SD*	*+ 2SD*
Model 1B: Proportion with schoolwork pressure	294,426	188	43	0.794	0.229	0.001	−6.574	14.612	−10.574	0.005	0.1360	0.1404	0.1449	0.1496	0.1543
Model 2B: Living with both parents	291,338	186	43	−0.968	0.249	<0.001	−2.675	18.490	−6.675	0.036	0.1549	0.1497	0.1446	0.1396	0.1348
Model 3B: Average time spent on internet use	131,287	81	31	0.240	0.060	<0.001	−9.969	9.601	−13.969	0.001	0.1313	0.1439	0.1574	0.1720	0.1875
Model 4B: Proportion with obesity	294,426	188	43	2.701	0.310	<0.001	−6.190	14.996	−10.190	0.006	0.1375	0.1415	0.1457	0.1499	0.1543

	Girls														
	Sample size			Model B country-year-level estimates			Model fit change relative to Model A				Predicted country proportions of high psychological complaints by values of predictors				
	*i*	*t*	*c*	*B*	*SE*	*p*	∆AIC	∆BIC	∆deviance	*p*	*−2 SD*^b^	*−1 SD*	0	*+ 1SD*	*+ 2SD*
Model 1B: Proportion with schoolwork pressure	315,881	188	43	1.515	0.201	<0.001	−31.782	−10.455	−35.782	<0.001	0.2373	0.2544	0.2723	0.2910	0.3105
Model 2B: Living with both parents	312,667	186	43	−1.160	0.293	<0.001	−1.674	19.631	−5.674	0.059	0.2889	0.2800	0.2712	0.2626	0.2542
Model 3B: Average time spent on internet use	139,081	81	31	0.363	0.049	<0.001	−32.599	−12.913	−36.599	<0.001	0.2256	0.2617	0.3012	0.3440	0.3894
Model 4B: Proportion with obesity	315,881	188	43	12.448	0.337	<0.001	−18.598	2.729	−22.598	<0.001	0.2394	0.2561	0.2735	0.2917	0.3105

*i* individuals, *t* years within countries, *c* countries, *B* logit coefficient, *SE* standard error, *p* *p*-value, *AIC* Akaike Information Criterion, *BIC* Bayesian Information Criterion, *SD* standard deviation. All model estimates can be found in the Supplementary (Tables S1-S4)

^a^Country-year-level *SDs* of boys’ cluster-mean centered proportion with schoolwork pressure, proportion living with both parents, average time spent on internet use, and proportion with obesity were 0.0466, 0.0418, 0.4412, and 0.0125, respectively

^b^Country-year-level *SDs* of girls’ cluster-mean centered proportion with schoolwork pressure, proportion living with both parents, average time spent on internet use, and proportion with obesity were 0.0610, 0.0379, 0.5393, and 0.0072, respectively

#### High schoolwork pressure and psychological complaints

Table 4 shows that adding high schoolwork pressure to the model improved model fit according to all fit indices, except for the BIC value for boys. Within a country, increases in the proportion of boys and girls with high schoolwork pressure were associated with increases in the proportion of boys and girls with high psychological complaints. The association was stronger for girls than for boys (*z* = 2.369, *p* = 0.018). Comparing years where the country proportion of girls with high schoolwork pressure was very low with years where this was very high (i.e., two standard deviations below versus above the country mean), the estimated country proportions of girls with high psychological complaints were 0.237 and 0.311 in the respective years. Among boys, these proportions ranged from 0.136 to 0.154 (Table 4).

#### Family structure and psychological complaints

For boys, adding family structure to the model improved model fit according to the AIC and deviance, but for girls only AIC improved (Table 4). The model estimates show that decreases within a country in the proportion of both girls and boys living with both parents were associated with increases in the proportion of girls and boys with high psychological complaints. The strength of this association did not differ significantly between girls and boys (*z* = 0.499, *p* = 0.618). Comparing years where the country proportion of girls living with both parents was very high with years where this was very low, the estimated country proportions of girls with high psychological complaints were 0.254 and 0.289 for the respective years. For boys, these proportion were 0.135 and 0.155 (Table 4).

#### Internet hours and psychological complaints

Table 4 shows that according to almost all fit indices adding internet hours to the model improved model fit (although this was not the case for the BIC value for the boys). Increases in the country average of time spent on internet use by girls and boys were associated with increases in the proportion of girls and boys with high psychological complaints. The strength of this association did not differ significantly between boys and girls (*z* = 1.586, *p* = 0.113). Comparing years where the country average in girls’ time spent on internet was very low with years where this was very high, the estimated country proportions of girls with high psychological complaints were 0.226 and 0.389 for these years. For boys, these proportion ranged between 0.131 and 0.188 (Table 4).

#### Obesity and psychological complaints

Adding obesity to the model improved model fit according to most fit indices (Table 4). Increases in countries’ proportion of girls and boys with obesity were associated with increases in countries’ proportion of girls and boys reporting high psychological complaints. This association was stronger among girls than among boys (*z* = 21.270, *p* < 0.001). Comparing years where the country proportion of girls with obesity was very low with years where this was very high, the estimated country proportions of girls with high psychological complaints were 0.239 and 0.311 in the respective years. Among boys, these proportions ranged from 0.138 to 0.154 (Table 4).

#### Final model

**Fig. 1:**
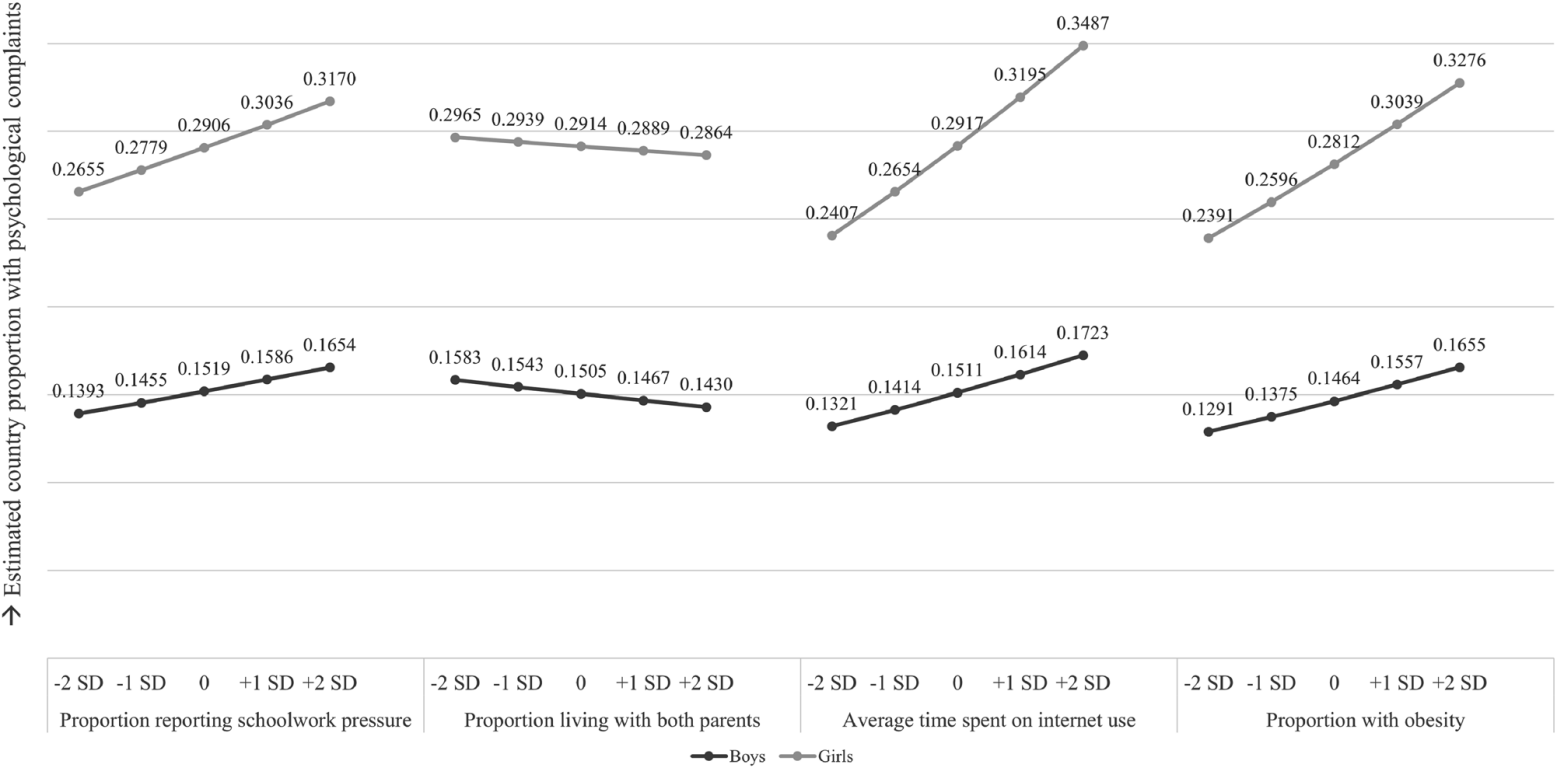
Estimated country proportions of high psychological complaints according to estimates from the multivariate model, by values of predictors and gender

Figure 1 illustrates the predicted country proportions of high psychological complaints by values of the predictors according to the estimates of the model where all four predictors were included simultaneously. In general, associations became smaller than in previous models, but most of the earlier found associations were still observed (Supplementary Table S5). Only the proportions of boys and girls living with both parents were not associated anymore with the proportions of boys and girls with high psychological complaints. However, post-hoc power analyses showed that there is low power to detect a significant effect of family structure given its effect size, the sample size, and/or covariates in the model. Should there be an effect of family structure, then Fig. 1 suggests that the strength of this effect is relatively small. Furthermore, Fig. 1 indicates that country-level changes in the average time spent on internet use and proportion with obesity and their associations with changes in psychological complaints proportions were about equally as strong. These associations appeared somewhat stronger than the association between changes in the proportion of adolescents with schoolwork pressure and psychological complaints. In addition, in the multivariate model, the association between the proportion of adolescents with obesity and the proportion of adolescents with psychological complaints was stronger among girls than among boys (*z* = 9.372, *p* < 0.001). The other associations did not differ by gender.

### Sensitivity Analyses

A set of further analyses examined whether these findings were robust to schoolwork pressure, family structure, and self-report obesity at the individual level. These predictors were significantly associated with a higher probability of high psychological complaints in both boys and girls (results not shown). Although effect sizes became smaller when controlling for these individual-level covariates, all earlier found associations remained, except for the association between the proportion of boys with schoolwork pressure and the proportion of boys with high psychological complaints.

Additional analysis using the 70^th^ and 90^th^ centile as cut-off value for high psychological complaints showed that in general estimates from these alternative models did not significantly differ from the initial 80^th^ centile models (based on *z*-tests, results not shown). Only the obesity associations were different: the higher the cut-off value for psychological complaints, the stronger the association between the proportion of boys and girls with obesity and the proportion of boys and girls with psychological complaints was.

## Discussion

While the impact of schoolwork pressure, family structure, internet use, and obesity on mental health problems has been studied extensively at the individual level, their impact at the societal level has rarely been explored. By studying associations between these phenomena at the country level from 2002 to 2018 within 43 European and North American countries, the present internationally-comparative study advances current knowledge on national-level drivers of adolescent mental health problems.

### Findings

Within the study period, psychological complaints increased more strongly among girls than boys. Furthermore, schoolwork pressure, the average time spent on internet, and obesity were generally rising, while proportions of adolescents living with both parents in one house were generally declining. Findings showed that within countries, increases in proportions of adolescents reporting high schoolwork pressure, proportions of adolescents with obesity, and average time spent on internet use were independently associated with increases in proportions of adolescents reporting high psychological complaints. The multivariate model with all four predictors included simultaneously suggests that national-level increases in obesity and time spent on internet use were about equally strongly related to increases in psychological complaints, and both seemed to explain trends in psychological complaints more strongly than trends in schoolwork pressure. Furthermore, the observed associations between trends appeared in both girls’ and boys’ samples. However, based on the multivariate model, increases in the proportions of adolescents with obesity were more strongly related to increases in proportions of high psychological complaints in girls’ than in boys’ samples. Decreases in proportions of boys and girls living with both parents were also associated with increases in proportions of boys and girls with psychological complaints, but these associations disappeared when controlling for trends in schoolwork pressure, obesity, and internet use in the multivariate model.

With regards to trends found for obesity, it could be argued that the co-occurrence of increases in national-level obesity and psychological complaints is due the fact that adolescents who are overweight or obese are more likely to experience psychological complaints (Whitehead et al., 2017), and therefore national-level increases in adolescent obesity rates might increase the proportion of adolescents with high psychological complaints. However, the observed country-level effects of obesity were robust to individual-level obesity (Brincks et al., 2017). This finding suggests that the revealed effects of obesity also reflect societal effects that are independent of obese adolescents’ individual susceptibility to psychological complaints. High obesity rates may drive the implementation of targeted health policies and prevention programs aimed at reducing and preventing obesity. When such policies focus on individual responsibility and lifestyle choices, rather than addressing wider structural determinants of obesity, they may inadvertently contribute to the stigmatization of overweight (Brewis et al., 2011), and could make (young) people more concerned with their own physical appearance (Hill et al., 2021). This may, in turn, lead to a society with more adolescents facing mental health issues (Brewis et al., 2018). Furthermore, results suggest that girls were more susceptible to the effect of high obesity rates within society than boys. This indicates that girls are not only more sensitive to experience mental health problems in response to individual stressors (Hankin et al., 2007), but also in response to specific societal stressors, such as exposure to obesity and possible associated weight-based stigmatization.

In addition, although an increasing number of studies show no or a very small association between internet use and mental health problems at the individual level (Appel et al., 2020; Meier & Reinecke, 2021; Odgers & Jensen, 2020), the present findings indicates that nevertheless, at the national level, increases in internet use and in the percentages of both boys’ and girls’ mental health problems were associated. This could imply that it is not necessarily adolescent’s individual engagement with internet that affects their mental health problems, but rather the wider online culture in which they grow up. High levels of internet use may reflect cultures where it is common to share and gather information with and about peers or others on social media, and this information is typically biased toward positivity (Lee et al., 2017). Adolescents who grow up in a digital landscape characterized by unrealistic portrayals of others may be prone to more negative self-evaluation and social comparison, ultimately harming their mental health (Verduyn et al., 2017). It should be noted, however, that in these analyses it was not possible to control for internet use at the individual level, which would provide more accurate estimates of the effect of internet use at the societal level (Brincks et al., 2017). However, it is expected that the association between national-level trends in internet use and psychological complaints is less affected by individual-level behaviors than the other included national-level predictors, because review studies show a nonexistent or small association between individual-level internet use and mental health problems (Appel et al., 2020; Meier & Reinecke, 2021; Odgers & Jensen, 2020), although other research contests this negligible association (Twenge et al., 2022).

Next to trends in obesity and internet use, national-level increases in schoolwork pressure were also associated with increases in psychological complaints. However, this association was robust to the inclusion of schoolwork pressure at the individual level for girls only. This implies that societies where schoolwork pressure is salient may pose a risk to the mental health of particularly girls. Possibly, increases in schoolwork pressure at the national level are reflective of a changing, more stressful society: a society that is more competitive, meritocratic, and in which the level of perfectionism is high (Curran & Hill, 2019). This societal context, where demands and expectations are high, likely increases the vulnerability to mental health problems. That this is especially true for girls is in line with the general notion that girls exhibit greater levels of mental health problems than boys in response to stressors, particularly to achievement stressors (De Looze et al., 2020; Hankin et al., 2007). Correspondingly, research proposes that higher learning intensity (i.e., quantity and complexity of learning tasks completed by a student) might be more detrimental for adolescent girls’ mental health compared to boys’, especially in wealthier nations (Rudolf & Bethmann, 2023). Trends in boys’ schoolwork pressure and psychological complaints were no longer associated when controlled for the association at the individual level. This implies that although schoolwork pressure strengthened the probability of experiencing high levels of psychological complaints in boys, there was no additional societal effect of schoolwork pressure at the national level on the mental health of boys.

Another finding worth mentioning is that the ICCs of psychological complaints indicated that only a very small percentage of the variance in psychological complaints was explained by country-level factors that changed over time, although this is not unusual for repeated individual-level cross-sectional data (Schmidt-Catran & Fairbrother, 2016; Tormos et al., 2017). This was because the largest share of the variance in psychological complaints was explained by individual-level characteristics. The observed associations in the present study, which were examined at the country-year level, therefore only explain a small part of the total variance in adolescents’ psychological complaints. Nevertheless, these findings are meaningful as the aim of present study was to explain national-level mental health trends rather than explaining total variance in adolescent mental health problems.

In addition, the associations between the national-level developments and psychological complaints were robust to confounding of each other (except for family structure), and, furthermore, to income inequality and economic performance. Despite this multivariate approach, it could be that these associations are explained by unobserved correlated trends. More specifically, societal factors such as the level of individualism, post-materialism, and meritocracy, likely play a role in explaining adolescent mental health trends as well. Cultures where these values prevail may place heavy demands on adolescents’ individual responsibility and self-reliance (Curran & Hill, 2022), taxing their mental health. Alternatively, it has been posited that increasing awareness of mental health problems in Western societies may lead adolescents with mild distress to become more inclined to identify with and report mental health problems (Foulkes & Andrews, 2023).

### Implications

Overall, these findings imply that not only processes that take place at the individual level, but also at the national level, may impact the mental health of adolescents. This highlights the importance of policies which target broader societal and structural systems which impact on individuals, rather than focusing solely on vulnerabilities at the individual level. In relation to obesity, discussing individual behavior changes necessary to prevent or overcome overweight and obesity are of crucial importance. Still, clinical and public health practitioners, policymakers, as well as researchers may want to reflect how public health messages around adolescent obesity are framed and promoted. It may be important to recognize the role of the environment in promoting obesity, for example through the ready availability and promotion of high fat, high sugar foods and targeting toward young people. Similarly, the results on the association between increases in schoolwork pressure and psychological complaints seem to suggest that education systems which prioritize academic achievement and success over other outcomes may be harmful to young people’s mental health. As a response to this, strategies may need to be developed that increase the resilience of adolescents in dealing with the pressure from schoolwork. At the same time, and in order to prevent adolescents from becoming solely responsible for dealing with this societal phenomenon, a broader societal discussion on the desirability of this increasing focus on academic achievement and (policy) changes to redress this, seems warranted. Furthermore, findings from this study reveal processes before the COVID-19 outbreak, that was declared a pandemic in March 2020. By identifying trends in and drivers of psychological complaints before the pandemic, the present study facilitates future research aimed at understanding changes in adolescent mental health problems during and since the pandemic.

### Strengths and Limitations

The present study includes nationally representative data of adolescents from many countries across a time period of almost two decades. It also combined data from different sources and used advanced statistical techniques to study the possible impact of societal developments that have characterized recent decades on adolescent mental health problems. Alongside these strengths, several limitations should be considered. First, the generalizability of these findings is limited to developed Western countries. Second, the number of countries and repeated measures yielded limited power to test the associations between all four predictors and high psychological complaints in the final multivariate model. Therefore, replicating the final model with more countries and/or more repeated measures is required to consolidate the finding that trends in family structure and psychological complaints were not related when controlling for other trends. Nevertheless, findings from Fig. 1 suggest that the potential effect of family structure would be small in magnitude. Third, (for some countries) observations on average time spent on internet, obesity, and income inequality were measured in other years than the HBSC survey rounds. Although using data from other years maximized the number of observations and thus power, it may have affected the precision of the model estimates. Especially the analysis with internet use warrants replication since this analysis had the most unmatched survey years. Fourth, the models did not include time as additional control variable due to multicollinearity. Although adding time could provide more precise estimations of associations between two time-varying phenomena within countries (Cosma et al., 2020), in some cases it could also distort such associations (L. P. Wang & Maxwell, 2015). Fifth, a more specific measure than time spent on internet use, that distinguishes different types of or addiction-like internet or social media use (Boer et al., 2022; Schønning et al., 2020), could improve current insights into the association with mental health and the strength of potential harmful effects of internet-related behaviors. Sixth, although the use of dichotomous measures of schoolwork pressure and psychological complaints provides insight into more severe levels of these constructs and their associations, dichotomizing implies some loss of information. Seventh, it must be acknowledged that the measure used to capture gender (“Are you a boy or girl”) is not inclusive and does not capture adolescents that might be gender diverse. Future studies would need to use a more gender inclusive measure in order to capture the time trends in gender minority adolescents.

## Conclusion

There are few studies that address the possible drivers of trends in adolescent mental health problems in adolescents. By examining four potential societal sources of national-level changes in adolescent psychological complaints between 2002 and 2018 across 43 countries, the present study aims to fill this gap. Findings imply that national contexts with highly prevalent internet use or obesity challenge the mental health of both boys and girls, and that national contexts with high schoolwork pressure pose a risk to the mental health of girls in particular. Such environments may reinforce a competitive context with societal pressure to be as good as peers appear online, to stay or become physically fit, and to succeed at school. This pressure to be successful across several major life domains likely taxes adolescents’ mental health. Results from this study underline that not only processes at the individual level, but also societal processes may impact adolescent mental health problems. Furthermore, they emphasize the importance of public health policies addressing risk factors for mental health at the societal level rather than focusing exclusively on individual-level preventive strategies.

## Supplementary information

Supplementary Information

## Footnotes

1 Through the survey years, several measures related to internet use, specifically on electronic media communication (EMC), were included in the HBSC study. However, these measures differ substantially by survey year and hence are not suited for trend analyses. Therefore, the external data was selected to indicate countries’ average time adolescents spend on internet use.

2 The HBSC-study includes measures related to obesity, namely self-reported weight and height. However, because these assessments are prone to bias, external data was selected to identify countries’ proportion of adolescents with obesity.

3 In this study, splitting the sample into boys and girls subsamples was required to test moderation instead of modelling an interaction parameter, because for correct model specification, higher level predictors (e.g., proportion with high schoolwork pressure at the country-year level), should not be multiplied with lower level predictors (e.g., gender at the individual level).
