# Supplementary material for: National-Level Schoolwork Pressure, Family Structure, Internet Use, and Obesity as Drivers of Time Trends in Adolescent Psychological Complaints Between 2002 and 2018
Source: J Youth Adolesc. 2023 Jun 22;52(10):2061–77. doi: 10.1007/s10964-023-01800-y (PMC10371956; doi:10.1007/s10964-023-01800-y)
Supplement: Supplementary file 1 — Supplementary Information [file 10964_2023_1800_MOESM1_ESM.docx]

**National-Level Schoolwork Pressure, Family Structure, Internet Use, and Obesity as Drivers of Time Trends in Adolescent Psychological Complaints Between 2002 and 2018**

*Journal of Youth and Adolescence*

# **Supplementary Material**

**Figure S1**

*Analytical model*

*
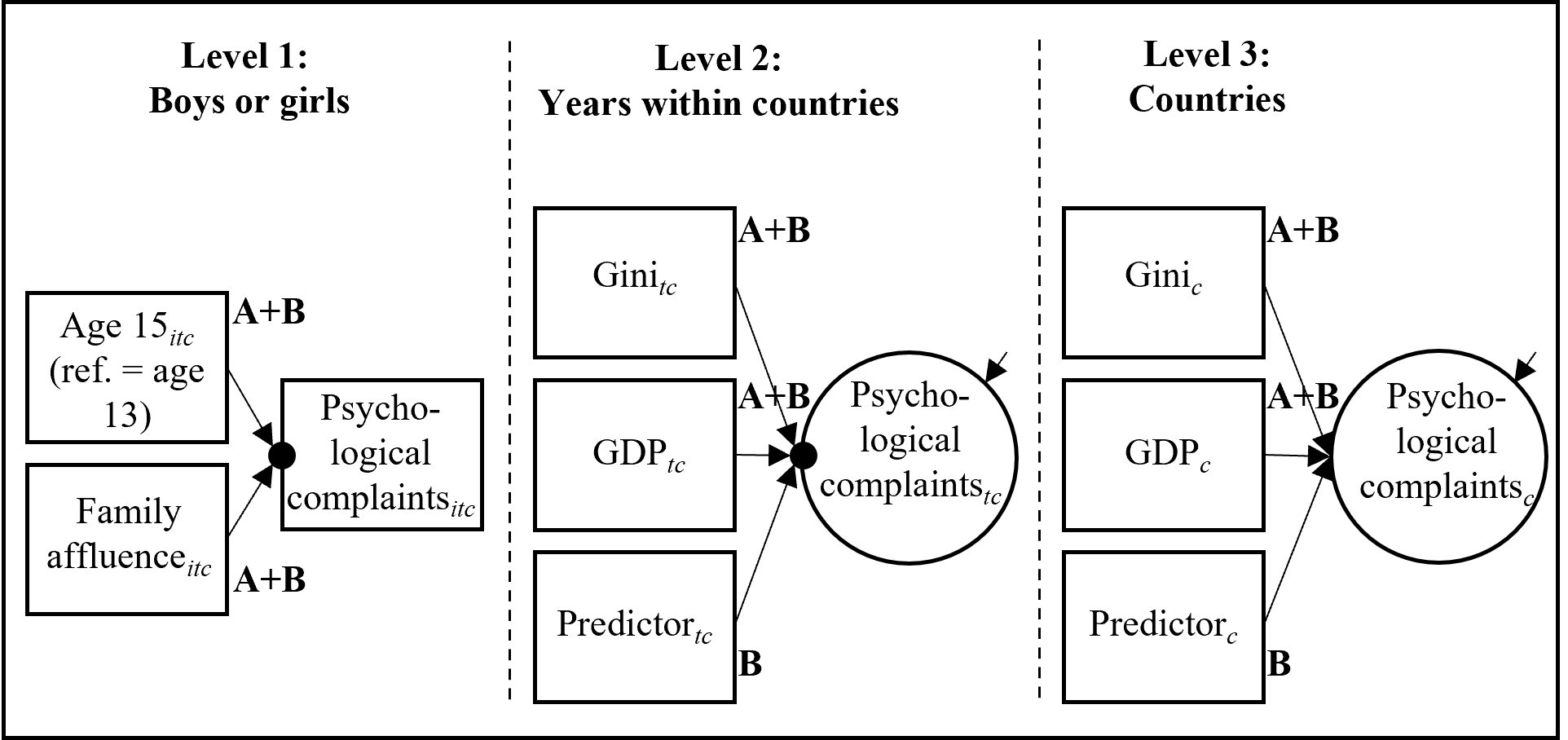
*

*Note.* White squares denote observed variables; Black circles indicate random intercepts that is referred to as the psychological complaints outcome at the second and third level, denoted by white circles;; Variables with subscript *itc* denote the values of the respective variable for individual *i* within country-year *t* within country *c*; Variables with subscript *tc* denote the values of the respective variable for country-year *t* within country *c,* centered by the respective country level mean; Variables with subscript *c* denote the values of the respective variable for country *c,* centered by the respective grand mean; Predictor = proportion with schoolwork pressure (M1A,B), proportion living with both parents (M2A,B), average internet hours per day (M3A,B), or proportion with obesity (M4A,B).

**Figure S2**

*Proportion of boys and girls with high psychological complaints 2002-2018, by country*

**Figure S3**

*Proportion of boys and girls with high schoolwork pressure 2002-2018, by country*

**Figure S4**

*Proportion of boys and girls living with both parents in one house 2002-2018, by country*

**Figure S5**

*Boys’ and girls’ average time (hours) spent on internet per day 2012-2018, by country*

**Figure S6**

*Proportion of boys and girls with obesity 2002-2016, by country*

**Figure S7**

*Income inequality (Gini index, 0 = equal, 100 = unequal) 2002-2018, by country*

**Figure S8**

*Economic performance (Gross Domestic Product divided by midyear population in US dollars) 2002-2018, by country*

| **Table S1**  *Three level logistic regression, high psychological complaints* | | | | | | | | | | | | |
| --- | --- | --- | --- | --- | --- | --- | --- | --- | --- | --- | --- | --- |
|  | **M1a** |  |  |  |  |  | **M1b** |  |  |  |  |  |
|  | **boys** |  |  | **girls** |  |  | **boys** |  |  | **girls** |  |  |
|  | ***B*** | ***SE*** | ***p*** | ***B*** | ***SE*** | ***p*** | ***B*** | ***SE*** | ***p*** | ***B*** | ***SE*** | ***p*** |
| *Level 1* |  |  |  |  |  |  |  |  |  |  |  |  |
| Age 15 (ref. = age 13) | 0.139 | 0.010 | < 0.001 | 0.326 | 0.008 | < 0.001 | 0.139 | 0.010 | < 0.001 | 0.326 | 0.008 | < 0.001 |
| Family affluence | -0.053 | 0.003 | < 0.001 | -0.054 | 0.002 | < 0.001 | -0.053 | 0.003 | < 0.001 | -0.054 | 0.002 | < 0.001 |
| *Level 2* |  |  |  |  |  |  |  |  |  |  |  |  |
| Income inequality (Gini) | -0.010 | 0.009 | 0.278 | -0.025 | 0.011 | 0.019 | -0.010 | 0.009 | 0.264 | -0.022 | 0.010 | 0.028 |
| Economic performance (GDP) | 0.308 | 0.052 | < 0.001 | 0.524 | 0.061 | < 0.001 | 0.296 | 0.051 | < 0.001 | 0.437 | 0.059 | < 0.001 |
| Proportion schoolwork pressure |  |  |  |  |  |  | 0.794 | 0.229 | 0.001 | 1.515 | 0.201 | < 0.001 |
| *Level 3* |  |  |  |  |  |  |  |  |  |  |  |  |
| Income inequality (Gini) | -0.020 | 0.013 | 0.135 | -0.014 | 0.012 | 0.264 | -0.021 | 0.013 | 0.087 | -0.015 | 0.011 | 0.166 |
| Economic performance (GDP) | -0.046 | 0.025 | 0.073 | -0.030 | 0.024 | 0.205 | -0.041 | 0.024 | 0.088 | -0.031 | 0.021 | 0.148 |
| Proportion schoolwork pressure |  |  |  |  |  |  | 1.091 | 0.295 | < 0.001 | 1.247 | 0.241 | < 0.001 |
| **Random parameters** | **Est.** |  |  | **Est.** |  |  | **Est.** |  |  | **Est.** |  |  |
| Variance level 2 (country-year) | 0.035 |  |  | 0.056 |  |  | 0.033 |  |  | 0.046 |  |  |
| Variance level 3 (country) | 0.134 |  |  | 0.110 |  |  | 0.121 |  |  | 0.087 |  |  |
| **Fit statistics** | **Est.** |  |  | **Est.** |  |  | **Est.** | **Difference A** | | **Est.** | **Difference A** | |
| Free parameters | 9 |  |  | 9 |  |  | 11 | 2 |  | 11 | 2 |  |
| AIC | 244236.038 |  |  | 363281.518 |  |  | 244229.464 |  |  | 363249.737 |  |  |
| BIC | 244331.373 |  |  | 363377.486 |  |  | 244345.985 |  |  | 363367.031 |  |  |
| Deviance | 244218.038 |  |  | 363263.518 |  |  | 244207.464 |  |  | 363227.737 |  |  |
| χ^2^ difference |  |  |  |  |  |  | 10.574 |  |  | 35.782 |  |  |
| *p* χ^2^ difference |  |  |  |  |  |  | 0.005 |  |  | < 0.001 |  |  |
| **Sample size** | ***n*** |  |  | ***n*** |  |  | ***n*** |  |  | ***n*** |  |  |
| Adolescents | 294426 |  |  | 315881 |  |  | 294426 |  |  | 315881 |  |  |
| Country-years | 188 |  |  | 188 |  |  | 188 |  |  | 188 |  |  |
| Countries | 43 |  |  | 43 |  |  | 43 |  |  | 43 |  |  |
| *** *p* < 0.001; ** *p* < 0.01; * *p* < 0.05; *B* = logit coefficient; *SE* = standard error; Est. = estimate; AIC = Akaike Information Criterion; BIC = Bayesian Information Criterion; χ^2^ = Chi-square. | | | | | | | | | | | | |

| **Table S2**  *Three level logistic regression, high psychological complaints* | | | | | | | | | | | | |
| --- | --- | --- | --- | --- | --- | --- | --- | --- | --- | --- | --- | --- |
|  | **M2a** |  |  |  |  |  | **M2b** |  |  |  |  |  |
|  | **boys** |  |  | **girls** |  |  | **boys** |  |  | **girls** |  |  |
|  | ***B*** | ***SE*** | ***p*** | ***B*** | ***SE*** | ***p*** | ***B*** | ***SE*** | ***p*** | ***B*** | ***SE*** | ***p*** |
| *Level 1* |  |  |  |  |  |  |  |  |  |  |  |  |
| Age 15 (ref. = age 13) | 0.137 | 0.011 | < 0.001 | 0.327 | 0.008 | < 0.001 | 0.137 | 0.011 | < 0.001 | 0.327 | 0.008 | < 0.001 |
| Family affluence | -0.053 | 0.003 | < 0.001 | -0.055 | 0.002 | < 0.001 | -0.053 | 0.003 | < 0.001 | -0.055 | 0.002 | < 0.001 |
| *Level 2* |  |  |  |  |  |  |  |  |  |  |  |  |
| Income inequality (Gini) | -0.010 | 0.009 | 0.260 | -0.027 | 0.011 | 0.014 | -0.007 | 0.009 | 0.437 | -0.023 | 0.011 | 0.038 |
| Economic performance (GDP) | 0.305 | 0.053 | < 0.001 | 0.516 | 0.062 | < 0.001 | 0.274 | 0.052 | < 0.001 | 0.477 | 0.062 | < 0.001 |
| Proportion living with both parents |  |  |  |  |  |  | -0.968 | 0.249 | < 0.001 | -1.160 | 0.293 | < 0.001 |
| *Level 3* |  |  |  |  |  |  |  |  |  |  |  |  |
| Income inequality (Gini) | -0.019 | 0.013 | 0.137 | -0.013 | 0.012 | 0.281 | -0.019 | 0.013 | 0.141 | -0.013 | 0.012 | 0.268 |
| Economic performance (GDP) | -0.045 | 0.025 | 0.076 | -0.028 | 0.023 | 0.220 | -0.043 | 0.025 | 0.090 | -0.024 | 0.023 | 0.292 |
| Proportion living with both parents |  |  |  |  |  |  | 0.205 | 0.292 | 0.483 | 0.404 | 0.243 | 0.097 |
| **Random parameters** | **Est.** |  |  | **Est.** |  |  | **Est.** |  |  | **Est.** |  |  |
| Variance level 2 (country-year) | 0.036 |  |  | 0.057 |  |  | 0.034 |  |  | 0.055 |  |  |
| Variance level 3 (country) | 0.132 |  |  | 0.105 |  |  | 0.132 |  |  | 0.103 |  |  |
| **Fit statistics** | **Est.** |  |  | **Est.** |  |  | **Est.** | **Difference A** | | **Est.** | **Difference A** | |
| Free parameters | 9 |  |  | 9 |  |  | 11 | 2 |  | 11 | 2 |  |
| AIC | 240796.743 |  |  | 359314.255 |  |  | 240794.068 |  |  | 359312.580 |  |  |
| BIC | 240891.983 |  |  | 359410.131 |  |  | 240910.472 |  |  | 359429.762 |  |  |
| Deviance | 240778.743 |  |  | 359296.255 |  |  | 240772.068 |  |  | 359290.580 |  |  |
| χ^2^ difference |  |  |  |  |  |  | 6.675 |  |  | 5.675 |  |  |
| *p* χ^2^ difference |  |  |  |  |  |  | 0.036 |  |  | 0.059 |  |  |
| **Sample size** | ***n*** |  |  | ***n*** |  |  | ***n*** |  |  | ***n*** |  |  |
| Adolescents | 291338 |  |  | 312667 |  |  | 291338 |  |  | 312667 |  |  |
| Country-years | 186 |  |  | 186 |  |  | 186 |  |  | 186 |  |  |
| Countries | 43 |  |  | 43 |  |  | 43 |  |  | 43 |  |  |
| *** *p* < 0.001; ** *p* < 0.01; * *p* < 0.05; *B* = logit coefficient; *SE* = standard error; Est. = estimate; AIC = Akaike Information Criterion; BIC = Bayesian Information Criterion; χ^2^ = Chi-square. | | | | | | | | | | | | |

| **Table S3**  *Three level logistic regression, high psychological complaints* | | | | | | | | | | | | |
| --- | --- | --- | --- | --- | --- | --- | --- | --- | --- | --- | --- | --- |
|  | **M3a** |  |  |  |  |  | **M3b** |  |  |  |  |  |
|  | **boys** |  |  | **girls** |  |  | **boys** |  |  | **girls** |  |  |
|  | ***B*** | ***SE*** | ***p*** | ***B*** | ***SE*** | ***p*** | ***B*** | ***SE*** | ***p*** | ***B*** | ***SE*** | ***p*** |
| *Level 1* |  |  |  |  |  |  |  |  |  |  |  |  |
| Age 15 (ref. = age 13) | 0.161 | 0.015 | < 0.001 | 0.318 | 0.012 | < 0.001 | 0.161 | 0.015 | < 0.001 | 0.317 | 0.012 | < 0.001 |
| Family affluence | -0.056 | 0.004 | < 0.001 | -0.043 | 0.003 | < 0.001 | -0.056 | 0.004 | < 0.001 | -0.043 | 0.003 | < 0.001 |
| *Level 2* |  |  |  |  |  |  |  |  |  |  |  |  |
| Income inequality (Gini) | 0.022 | 0.024 | 0.355 | -0.014 | 0.031 | 0.639 | 0.027 | 0.021 | 0.199 | -0.003 | 0.023 | 0.912 |
| Economic performance (GDP) | 0.281 | 0.084 | 0.001 | 0.355 | 0.110 | 0.001 | 0.060 | 0.091 | 0.513 | -0.001 | 0.091 | 0.989 |
| Average time spent on internet |  |  |  |  |  |  | 0.240 | 0.060 | < 0.001 | 0.363 | 0.049 | < 0.001 |
| *Level 3* |  |  |  |  |  |  |  |  |  |  |  |  |
| Income inequality (Gini) | -0.032 | 0.017 | 0.068 | -0.025 | 0.016 | 0.111 | -0.035 | 0.018 | 0.047 | -0.029 | 0.016 | 0.068 |
| Economic performance (GDP) | -0.063 | 0.036 | 0.083 | -0.049 | 0.034 | 0.145 | -0.062 | 0.036 | 0.089 | -0.048 | 0.033 | 0.151 |
| Average time spent on internet |  |  |  |  |  |  | -0.076 | 0.155 | 0.625 | 0.049 | 0.136 | 0.718 |
| **Random parameters** | **Est.** |  |  | **Est.** |  |  | **Est.** |  |  | **Est.** |  |  |
| Variance level 2 (country-year) | 0.038 |  |  | 0.075 |  |  | 0.028 |  |  | 0.035 |  |  |
| Variance level 3 (country) | 0.140 |  |  | 0.102 |  |  | 0.145 |  |  | 0.117 |  |  |
| **Fit statistics** | **Est.** |  |  | **Est.** |  |  | **Est.** | **Difference A** | | **Est.** | **Difference A** | |
| Free parameters | 9 |  |  | 9 |  |  | 11 | 2 |  | 11 | 2 |  |
| AIC | 114884.521 |  |  | 166781.768 |  |  | 114874.552 |  |  | 166749.170 |  |  |
| BIC | 114972.588 |  |  | 166870.354 |  |  | 114982.189 |  |  | 166857.441 |  |  |
| Deviance | 114866.521 |  |  | 166763.768 |  |  | 114852.552 |  |  | 166727.170 |  |  |
| χ^2^ difference |  |  |  |  |  |  | 13.969 |  |  | 36.599 |  |  |
| *p* χ^2^ difference |  |  |  |  |  |  | 0.001 |  |  | < 0.001 |  |  |
| **Sample size** | ***n*** |  |  | ***n*** |  |  | ***n*** |  |  | ***n*** |  |  |
| Adolescents | 131287 |  |  | 139081 |  |  | 131287 |  |  | 139081 |  |  |
| Country-years | 81 |  |  | 81 |  |  | 81 |  |  | 81 |  |  |
| Countries | 31 |  |  | 31 |  |  | 31 |  |  | 31 |  |  |
| *** *p* < 0.001; ** *p* < 0.01; * *p* < 0.05; *B* = logit coefficient; *SE* = standard error; Est. = estimate; AIC = Akaike Information Criterion; BIC = Bayesian Information Criterion; χ^2^ = Chi-square. | | | | | | | | | | | | |

| **Table S4**  *Three level logistic regression, high psychological complaints* | | | | | | | | | | | | |
| --- | --- | --- | --- | --- | --- | --- | --- | --- | --- | --- | --- | --- |
|  | **M4a** |  |  |  |  |  | **M4b** |  |  |  |  |  |
|  | **boys** |  |  | **girls** |  |  | **boys** |  |  | **girls** |  |  |
|  | ***B*** | ***SE*** | ***p*** | ***B*** | ***SE*** | ***p*** | ***B*** | ***SE*** | ***p*** | ***B*** | ***SE*** | ***p*** |
| *Level 1* |  |  |  |  |  |  |  |  |  |  |  |  |
| Age 15 (ref. = age 13) | 0.139 | 0.010 | < 0.001 | 0.326 | 0.008 | < 0.001 | 0.139 | 0.010 | < 0.001 | 0.326 | 0.008 | < 0.001 |
| Family affluence | -0.053 | 0.003 | < 0.001 | -0.054 | 0.002 | < 0.001 | -0.053 | 0.003 | < 0.001 | -0.055 | 0.002 | < 0.001 |
| *Level 2* |  |  |  |  |  |  |  |  |  |  |  |  |
| Income inequality (Gini) | -0.010 | 0.009 | 0.278 | -0.025 | 0.011 | 0.019 | -0.010 | 0.009 | 0.274 | -0.025 | 0.010 | 0.012 |
| Economic performance (GDP) | 0.308 | 0.052 | < 0.001 | 0.524 | 0.061 | < 0.001 | 0.242 | 0.052 | 0.000 | 0.365 | 0.058 | < 0.001 |
| Proportion obesity |  |  |  |  |  |  | 2.701 | 0.310 | < 0.001 | 12.448 | 0.337 | < 0.001 |
| *Level 3* |  |  |  |  |  |  |  |  |  |  |  |  |
| Income inequality (Gini) | -0.020 | 0.013 | 0.135 | -0.014 | 0.012 | 0.264 | -0.028 | 0.012 | 0.021 | -0.017 | 0.012 | 0.139 |
| Economic performance (GDP) | -0.046 | 0.025 | 0.073 | -0.030 | 0.024 | 0.205 | -0.064 | 0.024 | 0.007 | -0.042 | 0.023 | 0.061 |
| Proportion obesity |  |  |  |  |  |  | 5.476 | 0.370 | < 0.001 | 3.817 | 0.237 | < 0.001 |
| **Random parameters** | **Est.** |  |  | **Est.** |  |  | **Est.** |  |  | **Est.** |  |  |
| Variance level 2 (country-year) | 0.035 |  |  | 0.056 |  |  | 0.034 |  |  | 0.049 |  |  |
| Variance level 3 (country) | 0.134 |  |  | 0.110 |  |  | 0.113 |  |  | 0.101 |  |  |
| **Fit statistics** | **Est.** |  |  | **Est.** |  |  | **Est.** | **Difference A** | | **Est.** | **Difference A** | |
| Free parameters | 9 |  |  | 9 |  |  | 11 | 2 |  | 11 | 2 |  |
| AIC | 244236.038 |  |  | 363281.518 |  |  | 244229.848 |  |  | 363262.921 |  |  |
| BIC | 244331.373 |  |  | 363377.486 |  |  | 244346.369 |  |  | 363380.215 |  |  |
| Deviance | 244218.038 |  |  | 363263.518 |  |  | 244207.848 |  |  | 363240.921 |  |  |
| χ^2^ difference |  |  |  |  |  |  | 10.190 |  |  | 22.598 |  |  |
| *p* χ^2^ difference |  |  |  |  |  |  | 0.006 |  |  | < 0.001 |  |  |
| **Sample size** | ***n*** |  |  | ***n*** |  |  | ***n*** |  |  | ***n*** |  |  |
| Adolescents | 294426 |  |  | 315881 |  |  | 294426 |  |  | 315881 |  |  |
| Country-years | 188 |  |  | 188 |  |  | 188 |  |  | 188 |  |  |
| Countries | 43 |  |  | 43 |  |  | 43 |  |  | 43 |  |  |
| *** *p* < 0.001; ** *p* < 0.01; * *p* < 0.05; *B* = logit coefficient; *SE* = standard error; Est. = estimate; AIC = Akaike Information Criterion; BIC = Bayesian Information Criterion; χ^2^ = Chi-square. | | | | | | | | | | | | |

| **Table S5**  *Three-level logistic regression, high psychological complaints* | | | | | | |
| --- | --- | --- | --- | --- | --- | --- |
|  | **boys** |  |  | **girls** |  |  |
|  | ***B*** | ***SE*** | ***p*** | ***B*** | ***SE*** | ***p*** |
| *Level 1* |  |  |  |  |  |  |
| Age 15 (ref. = age 13) | 0.164 | 0.016 | < 0.001 | 0.329 | 0.012 | < 0.001 |
| Family affluence | -0.056 | 0.004 | < 0.001 | -0.043 | 0.003 | < 0.001 |
| *Level 2* |  |  |  |  |  |  |
| Income inequality (Gini) | 0.034 | 0.020 | 0.092 | 0.012 | 0.021 | 0.580 |
| Economic performance (GDP) | 0.020 | 0.086 | 0.813 | -0.016 | 0.086 | 0.850 |
| Proportion schoolwork pressure | 1.088 | 0.478 | 0.023 | 1.024 | 0.419 | 0.015 |
| Proportion living with both parents | -0.713 | 0.519 | 0.169 | -0.323 | 0.888 | 0.716 |
| Average time spent on internet | 0.177 | 0.061 | 0.003 | 0.243 | 0.056 | < 0.001 |
| Proportion obesity | 5.822 | 0.585 | < 0.001 | 15.225 | 0.815 | < 0.001 |
| *Level 3* |  |  |  |  |  |  |
| Income inequality (Gini) | -0.020 | 0.014 | 0.171 | -0.019 | 0.013 | 0.145 |
| Economic performance (GDP) | -0.040 | 0.029 | 0.169 | -0.016 | 0.027 | 0.566 |
| Proportion schoolwork pressure | -0.695 | 0.456 | 0.128 | 0.512 | 0.449 | 0.254 |
| Proportion living with both parents | 1.239 | 0.580 | 0.033 | 0.435 | 0.587 | 0.459 |
| Average time spent on internet | 0.272 | 0.144 | 0.059 | 0.286 | 0.127 | 0.024 |
| Proportion obesity | 6.630 | 0.665 | < 0.001 | 1.576 | 0.520 | 0.002 |
| **Random parameters** | **Est.** |  |  | **Est.** |  |  |
| Variance level 2 (country-year) | 0.023 |  |  | 0.030 |  |  |
| Variance level 3 (country) | 0.081 |  |  | 0.063 |  |  |
| **Fit statistics** | **Est.** |  |  | **Est.** |  |  |
| Free parameters | 17 |  |  | 17 |  |  |
| AIC | 109143.141 |  |  | 160218.682 |  |  |
| BIC | 109308.862 |  |  | 160385.380 |  |  |
| Deviance | 109109.141 |  |  | 160184.682 |  |  |
| **Sample size** | ***n*** |  |  | ***n*** |  |  |
| Adolescents | 126538 |  |  | 134027 |  |  |
| Country-years | 78 |  |  | 78 |  |  |
| Countries | 30 |  |  | 30 |  |  |
| *** *p* < 0.001; ** *p* < 0.01; * *p* < 0.05; *B* = logit coefficient; *SE* = standard error; Est. = estimate; AIC = Akaike Information Criterion; BIC = Bayesian Information Criterion.; χ^2^ = Chi-square. | | | | | | |
